# Accuracy Study of Kato-Katz and Helmintex Methods for Diagnosis of Schistosomiasis Mansoni in a Moderate Endemicity Area in Sergipe, Northeastern Brazil

**DOI:** 10.3390/diagnostics13030527

**Published:** 2023-01-31

**Authors:** Daniel Lima Menezes, Carlos Thailan de Jesus Santos, Yvanna Louise Di Christine Oliveira, Vinícius Torres Castro Campos, Deborah Aparecida Negrão-Corrêa, Stefan Michael Geiger, José Rodrigo Santos Silva, Sona Jain, Luciana Maria Oliveira, Ricardo Toshio Fujiwara, Carlos Graeff-Teixeira, Silvio Santana Dolabella

**Affiliations:** 1 Postgraduate Program in Pharmaceutical Sciences, Federal University of Sergipe, São Cristóvão 49100-000, SE, Brazil; 2 Postgraduate Program in Parasite Biology, Federal University of Sergipe, São Cristóvão 49100-000, SE, Brazil; 3 Department of Morphology, Federal University of Sergipe, São Cristóvão 49100-000, SE, Brazil; 4 Department of Parasitology, Federal University of Minas Gerais, Belo Horizonte 31270-901, MG, Brazil; 5 Postgraduate Program in Industrial Biotechnology, Tiradentes University, Aracaju 49032-490, SE, Brazil; 6 Infectious Diseases Unit, Department of Pathology, Federal University of Espírito Santo, Vitória 29047-105, ES, Brazil

**Keywords:** *Schistosoma mansoni*, diagnostic, Kato-Katz, Helmintex, sensitivity, accuracy

## Abstract

Schistosomiasis is a neglected tropical disease (NTD) caused by blood flukes from the genus *Schistosoma.* Brazil hosts the main endemic area in the Americas, where *Schistosoma mansoni* is the only species causing the disease. Kato-Katz (KK) thick smear is the WHO recommended screening test for populational studies, but there is growing evidence for the sensitivity limitations associated with KK, especially in areas with low parasite loads. Helmintex (HTX) is another highly sensitive egg-detection method, based on the magnetic properties of *S. mansoni* eggs and their isolation in a magnetic field. The objective of this study is to evaluate both KK and HTX in a moderate endemic locality, Areia Branca, located in the municipality of Pacatuba, in the state of Sergipe in northeastern Brazil. From 234 individual fecal samples, two KK thick smears were prepared and evaluated for each sample. Similarly, 30 g of each fecal sample was processed by HTX protocol. Eggs were detected in 80 (34.18%) residents. Twenty-three (9.83%) samples were positive for eggs (only by KK), and 77 (32.91%) samples showed positive for eggs (only by HTX). Sensitivity, specificity, and accuracy estimates gave values of 28.75%, 100% and 75.64%, respectively, for KK, and 96.25%, 100% and 98.72% respectively, for HTX. The positive predictive value was 100% for both methods, while the negative predictive value was 72.99% for KK and 98.09% for HTX. Overall, HTX presented a superior performance compared to the one sample, two slides KK examination. The study confirms the role of HTX as a reference method for the definition of true-positive samples in comparative accuracy studies and its potential role in the late stages when the certification of schistosomiasis transmission interruption is required. Diagnostic tests are important tools for the elimination of this NTD, besides the effective implementation of safe water, basic sanitation, snail control, and the treatment of infected populations.

## 1. Introduction

Schistosomiasis is a parasitic infection caused by trematodes from the genus *Schistosoma*. Transmission takes place in aquatic environments contaminated with human feces containing schistosome eggs [1]. It is a neglected tropical disease that mainly affects populations struggling with poverty and the lack of adequate water, basic sanitation, and education. The expansion of transmission areas have recently been detected in urban and peri-urban areas, especially those with rapid and unplanned populational booms [2,3].

*Schistosoma* species are distributed mainly in Africa, Latin America, the Middle East and Southeastern Asia [4,5]. It is estimated that 252 million people are infected and more than 709 million from 78 countries and territories live under the risk of infection [6]. In Brazil, *Schistosoma mansoni* is the only species causing infections and schistosomiasis is still a public health concern considering the 102.259 DALY’s per 10^5^ inhabitants (Disability Adjusted Life Years) documented in 2016 [7]. Schistosomiasis is mainly endemic in the northeastern and southeastern regions [8].

Among the most susceptible groups are young men and adults between the ages of 16 and 25, mainly due to their greater exposure to contaminated water, living in housing in locations without basic sanitation and access to treated water, and recreational and work activities such as bathing and fishing [9].

Egg detection in fecal samples is required for confirmed diagnosis of schistosomiasis. The Kato-Katz (KK) thick smear method has been the recommended screening method because of its lower complexity and minimal operational costs [10]. Moreover, the prepared slides are easy to store at room temperature for months for a later examination by microscope [11,12,13]. Its disadvantages include the inability to analyze watery stools and the uneven distribution of eggs in fecal material, leading to false-negative outcomes [14,15]. Furthermore, especially in low endemic settings, KK lacks sensitivity and may underestimate the prevalence with prejudice for adequate guidance for control measures [15].

Helmintex (HTX) involves several concentration steps with the isolation of *S. mansoni* eggs from 30 g of feces, through their interaction with paramagnetic beads in a magnetic field. Seeding experiments in the laboratory have demonstrated that HTX is 100% sensitive for egg burdens higher than 1.3 eggs per gram of feces (EPG) [16,17]. As HTX includes a laborious and complex set of procedures, it is not adequate for the routine screening of populations in low endemicity areas, but it is a valuable reference method for comparative studies of diagnostic tests [18,19].

Our objective was to evaluate KK and HTX methods in moderate and low endemicity settings, especially in samples with low egg burden and where monitoring programs suggest a reduction in transmission. Moreover, we compared the potential of both tests to detect the infection at different ages and sex, since these variables may affect the results from diagnostic tests, especially due to variable risk for infection and successive reinfections. The results were further compared to a consolidated reference standard (CRS) composed of egg detection by either of the two methods.

## 2. Materials and Methods

### 2.1. Ethics Statement

All procedures used for this study were approved by the Research Ethics Committee of the Federal University of Sergipe (CAAE: 66074017.6.1001.5546) and the Federal University of Minas Gerais (CAAE no. 55239522.3.0000.5149). Parents/guardians and participating children were informed about the purpose and procedure of the study. Written informed consent was obtained from the parents/guardians of the children agreeing to participate in the study prior to enrolment. All human stool samples obtained in this study were coded and treated anonymously.

The participants were informed of their stool test results, and all of the infections were treated with praziquantel. The dosing utilized included 40 mg/kg for adults and 60 mg/kg for children, according to Brazilian technical regulations [20]. Individuals infected with *Ascaris lumbricoides*, *Trichuris trichiura* and hookworms were treated with a single dose of 400 mg albendazole.

### 2.2. Study Area and Population

**Figure 1:**
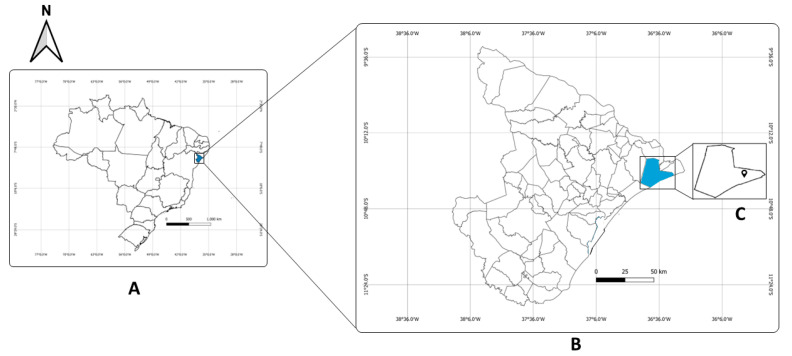
(**A**) Map of Brazil; (**B**) Map of the State of Sergipe (Brazil) showing Pacatuba municipality (**C**).

The survey was conducted in Areia Branca in the municipality of Pacatuba, Sergipe, Brazil (10°28′04.7″ S, 36°38′00.6″ W), located at a distance of 95 Km from Aracaju, the state capital (Figure 1). Situated next to the Betume River [21], the surveyed locality included 441 inhabitants [22]. Requirements for inclusion in the study were: (i) age > 6 years-old and (ii) collection of a fecal sample >30 g. Previous prevalence estimates from a national survey (Inquérito Nacional de Prevalência da Esquistossomose mansoni e Geo-helmintoses, INPEG) indicated that the municipality of Pacatuba is an endemic area with moderate prevalence [23], while the locality of Areia Branca showed a positivity index of 12.98% (positive egg results from KK examinations), according to data from the Brazilian National Schistosomiasis Control Program [23]. The age of the studied population varied from between six and 97 years, with an average of 35.5 ± 18.1 years. Among the 234 selected participants, 52 (22.2%) were between six and 20 years old, 160 (68.4%) were between 21 and 60 years old, and 22 (9.4%) were over 60 years old. A higher number of the volunteers were women (56.98%).

### 2.3. Collection of Biological Samples and Laboratory Procedures

**Figure 2:**
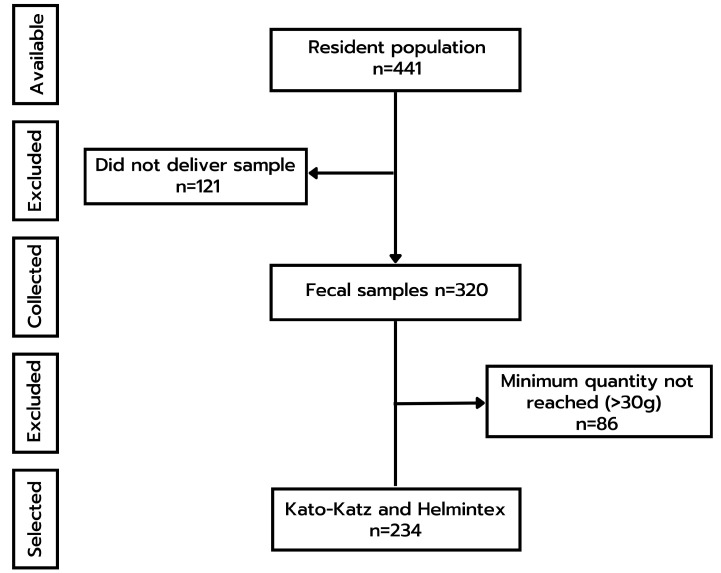
Flow chart showing the sample inclusion and exclusion criteria utilized in this study.

Large (1 L) plastic containers were distributed for sample collection and samples were processed in the laboratory by the KK and HTX methods (see flow chart, Figure 2). From a total of 441 residents, 320 individual fecal samples were collected, and 234 (73.1%) of these samples weighed >30 g. Results were individually reported back to the population together with treatment options and information on preventive measures.

A commercial KK kit, HelmTest^®^ (Biomanguinhos/FIOCRUZ, Rio de Janeiro, RJ, Brazil) was used, and two slides were prepared from each sample, as recommended by the World Health Organization [13] and many other authors [23,24]. A small portion of the stool sample to be examined was placed on a piece of toilet paper and pressed with a metal mesh [25]. The sieved fecal material was transferred to fill a 6 mm orifice on a plastic card placed on a glass slide made to contain approximately 42 mg of the sieved feces. After removing the card, the small amount of feces was covered with cellophane impregnated with malachite green and glycerin. The thick smear was obtained by pressing the slide with one finger. The screening of the smear under the microscope (100× magnification) was performed after 1 to 2 h. The number of EPG for each sample was established by the average egg counting from two slides, multiplied by 24.

HTX was performed according to the revised protocol as reported by Favero et al. [26] Briefly, 30 g of feces was fixed in 10% of Tween-20 with 70% ethanol (*v*/*v*) and filtered through a sieve with an aperture of 500 μm. After consecutive washing and sedimentation, the pellet was passed through two different sieves (apertures of 150 and 45 μm). The material retained on the last sieve (45 μm) was processed by the Ritchie method (1948), and 19 μL of paramagnetic particles (Bangs Labs, Fishers, IN, USA) was added to the final sediment. After incubation for 30 min, the microtubes containing the final sediment were placed on a magnet rack (Bangs Labs, Fishers, IN, USA) for 3 min, and the material that did not adhere to the wall of the microtube was discarded. The final pellet was stained with 3% ninhydrin for 15 min at 24 °C, placed on rectangular filter paper, and screened under a microscope at 100× magnification.

### 2.4. Statistical Analysis

A CRS was established using the positive egg identification by the KK and HTX methods [27]. The normality of distributions was verified with a Shapiro-Wilk test. The proportions of positive and negative results were compared with a McNemar test, considering CRS, and prevalence ratios were estimated from each of the test results. Positive (PPV) and negative (NPV) predictive values, sensitivity, specificity, and the receiver operating characteristic curve (ROC) were also calculated to access the accuracy of the diagnostic tests. The variation of discrete variables (age of individuals) was analyzed by the Wilcoxon test. Analysis and estimations were obtained by using RStudio 4.2.2 (Boston, MA, USA, EUA).

## 3. Results

Eggs were detected in 23 and 77 individual samples, by KK and HTX methods, respectively (Table 1). The analysis of the distribution of positive outcomes according to sex demonstrated that a higher probability for infection is associated with the male condition, as estimated from both KK (RP: 2.91) and HTX (RP: 1.79) data. No significant increased risk was associated with age group.

### 3.1. Performance of the Parasitological Methods for Schistosomiasis Diagnosis

The proportion of positive outcomes, as established by the CRS, was 34.2% (80/234), while it was 9.8% and 32.9%, respectively, when considering KK and HTX individually. Thus, 80 out of 234 individuals, as defined by CRS, were the “true positive samples” or “positive controls” (+C).

While only 23 out of 80 +C were detected by KK (28.8% sensitivity), HTX detected 77 out of 80 +C (96.2% sensitivity), resulting in accuracy estimations of 75.6% and 98.7% for KK and HTX, respectively (Table 2). Based on an analysis of ROC, there is a higher probability for detection of schistosomiasis for HTX (0.98) as compared to KK (0.64).

### 3.2. Classification According to Egg Burdens

**Figure 3:**
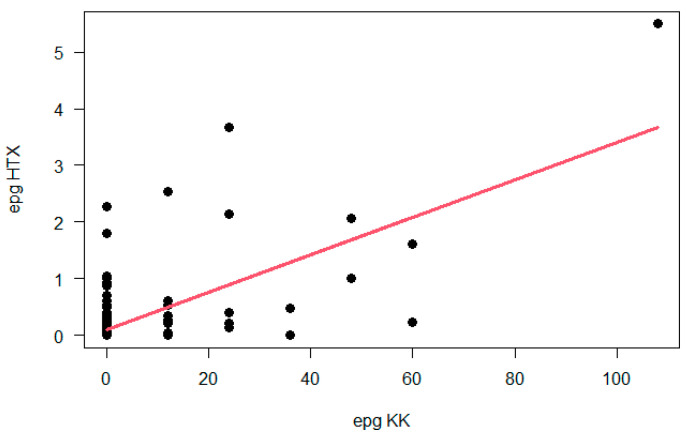
Case-to-case comparisons of the egg burden values estimated by the Kato-Katz and Helmintex methods. The correlation coefficient is 0.4336829.

According to the WHO, the egg burden should be accessed by KK to classify the infection intensity as light (from 1 to 99 EPG); moderate (100 to 399 EPG) and heavy (≥400 EPG) [13]. In Areia Branca, 95.7% (22/23) of positive samples presented light infections and only one resident (4.3%) presented higher EPG (108, average). The distribution and comparison of egg counts from KK and HTX is shown in Figure 3.

## 4. Discussion

Since the implementation of the Brazilian Schistosomiasis Control Program (*Programa de Controle da Esquistossomose*, PCE) there have been indications of decreased morbidity, reduced infection intensity and low prevalence [28,29]. Although most of the Brazilian endemic localities are close to achieving the goal of less than 1% of heavy infections (>400 EPG), there is still plenty of work to be carried out [13].

The many challenges to effectively reach the advanced stages of schistosomiasis morbidity and transmission control include the lack of sensitive diagnostic tests able to detect light asymptomatic infections [4,30]. These residual undetected and untreated infections keep contributing the eggs that are being eliminated into the environment. The test-treat strategy is essential for successful schistosomiasis control, especially in settings with low endemicity [31]. Moreover, the reduction of egg sources has an even more important role when we consider the low feasibility of sustained population control and the eradication of the snail hosts. Therefore, the development and adequate evaluation of diagnostic tests are essential for successful schistosomiasis control.

The history of exposure to water bodies for leisure or work activities has been well documented as risk determinants for schistosomiasis [32]. The present data shows an increased risk of infection for male individuals, who are more involved with fishing and agricultural activities in Areia Branca [33,34,35]. The absence of significant risk associated with age is also noteworthy, suggesting that all age groups are equally exposed to infection.

The distribution of egg burdens in the studied population shows that most individuals were eliminating very low egg numbers, except for a single participant with 108 EPG, which explains the large difference of positivity between results obtained by KK and HTX [18,36,37]. HTX was clearly superior to KK for detecting infections in Areia Branca.

Among other strategies to improve the accuracy of KK in low endemicity settings, increased numbers of thick smears have been used, but the few studies with direct KK-HTX comparison have failed to demonstrate that a bigger sampling can result in KK positivity equivalent to HTX positivity [19,38]. After comparing 14 KK smears (one fecal sample) with HTX, Oliveira and collaborators estimated KK sensitivity to be approximately 60% lower than HTX sensitivity for samples with less than 12 EPG [18]. Increasing the number of samples per individual to three, with two smears for each sample, KK was able to detect 40% of the positive samples, while HTX detected 84% of them [18].

Large numbers of slides were screened using a microscope, both resulting from an extended number of KK thick smears or from HTX, which are too laborious for routine examinations at the populational level. The sensitivity advantage of HTX over KK comes from an initial fecal volume that is 714 times larger in the case of HTX (30 g), when compared to KK (0.042 g). However, larger fecal volumes (in case of HTX) are associated with laborious and time-consuming procedures. There are indications that smaller amounts of feces may not be associated with a significant reduction in HTX sensitivity, even at low intensity areas [14], but modifications in the standard protocol require further standardization experiments [26].

Other diagnostic methods for *S. mansoni* are being tested (one of them is the detection of circulating cathode antigen in urine samples (POC-CCA)) as an alternative to the KK method. The POC-CCA is easy to use, of low cost, and has a shorter execution time and greater patient adherence in addition to having greater sensitivity compared to KK (29.1 vs. 3.6%, when trace observations were considered as positive) [39]. Despite the advantages, the POC-CCA can present false-positive results [40] and not present reproducibility with different batches used [38], and thus is not being recommended as a reliable diagnostic tool in areas of low endemicity [41].

In our study, the accuracy, sensitivity and specificity estimated from the present data demonstrate a superior performance of HTX, as compared to KK. HTX successfully detected 96.25% of CRS-true-positive samples, a positivity ratio 3.3 times higher than that obtained with KK (28.8%). The present study corroborates similar studies carried out in low endemicity regions [14,42]. It is also interesting to highlight that HTX showed greater sensitivity even when compared to polymerase chain reaction (PCR) [43].

Better accuracy was also indicated by ROC analysis. Similar large differences of sensitivity estimates for HTX as compared to KK were also reported by Oliveira and collaborators (84.4%) [18] and Magalhães and collaborators (63.8%) [19].

HTX yielded negative results in 3 KK-positive samples, resulting in a negative predictive value (NPV) of 98.09% for HTX vs. 72.9% for KK. Lindholz and collaborators reported similar differences with NPV of 100% for HTX vs. 67.2% for KK. The authors also found similar KK estimates for accuracy (71.1 vs. 75.64 in present study) and sensitivity (29.3% vs. 28.75%) [14].

As previously highlighted by other authors [14], egg counts are overestimated by KK as compared to HTX (Figure 3). It is also interesting to note that the endemicity setting in Areia Branca shows a dissociation between the prevalence (32.9%) and intensity of infections, with most egg burdens lower than 1 EPG. This dissociation may result from continuous but less than adequate control measures, which prevent more severe infections but does not effectively contribute to transmission elimination.

Moderate and low endemicity settings are defined by the WHO as areas with a prevalence between 10% and 50% and lower than 10%, respectively [13]. Areia Branca would be classified as a low endemicity area if the prevalence is based on KK results (9.82%) from this study. However, a more accurate prevalence is provided by HTX at 32.9%, upgrading Areia Branca as a moderate endemicity area. These discrepant findings highlight the limitations of the KK method for guiding late stages of schistosomiasis control with predominant light infections. Official 2021 data from the Pacatuba municipality registered prevalence estimates of 12.63% [22], which is close to the KK estimate of 9.82% in the present study, but also much lower than the HTX estimate of 32.9%. It is noteworthy that in Pacatuba, routine examinations are performed with only one thick smear, and not the two slides recommended by the WHO (as performed in the study), which poses further limitations to current screening protocols.

## 5. Conclusions

The evaluation of two diagnostic tests in a moderate endemicity area in Areia Branca, Pacatuba, Sergipe, northeastern Brazil has confirmed the limitations of KK and the potential contribution of HTX as a reference method to define true-positive samples. The development and adequate evaluation of diagnostic tests are pivotal for eliminating schistosomiasis as a public health problem, its transmission at a late stage, and the certification of the absence of transmission. The HTX method is currently under revision (by our research group) for improvements that will eventually decrease its laborious character, without reducing its striking sensitivity. Among the improvements, a more efficient sieving procedure may significantly decrease the time required for the detection of eggs.

## Figures and Tables

**Table 1:** Distribution of positive results by Kato-Katz and Helmintex according to sex and age.

Category	Variable	P (%)	N (%)	PR (CI 95%)	*p*-Value
KK vs. SEX	men	16 (15.5)	87 (84.5)	2.91 (1.24–6.80)	0.017
	women	7 (5.3)	124 (94.7)	1	
HTX vs. SEX	men	45 (43.7)	58 (56.3)	1.79 (1.23–2.60)	0.003
	women	32 (24.4)	99 (75.6)	1	
KK vs. AGE		42.0 ± 25.0	34.0 ± 27.0	-	0.442 *
HTX vs. AGE		35.0 ± 18.0	34.0 ± 35.0	-	0.543 *

Positive (P), Negative (N), Prevalence Ratio (PR), Confidence Intervals (CI), Kato-Katz (KK), Helmintex (HTX). Considered significant *p*-value: *p* ≤ 0.05. * No significant increased risk was associated with different ages.

**Table 2:** Performance indicators for the Kato-Katz and Helmintex methods.

	Sensitivity (%)	Specificity (%)	Accuracy (%)	PPV (%)	NPV (%)	AUC
KK	28.8	100.00	75.6	100.00	7.9	0.64
HTX	96.2	100.00	98.7	100.00	98.1	0.98

Kato-Katz (KK), Helmintex (HTX), Positive Predictive Value (PPV), Negative Predictive Value (NPV), Area Under the Curve (AUC), CI = 95%.

## Data Availability

Not applicable.
